# Validation of Standardized Nursing Languages for People Living with HIV Over 50

**DOI:** 10.1093/geroni/igaf122.4043

**Published:** 2025-12-31

**Authors:** Juh Shin, Sue Moorhead, Elizabeth Swanson, Mary Clarke, Cheryl Wagner, Karen Dunn Lopez, Nidhi Naidu, Hritik Majgaonkar

**Affiliations:** George Washington University, Ashburn, Virginia, United States; University of Iowa, Iowa City, Iowa, United States; University of Iowa Center of Nursing Classification and Clinical Effectiveness, Iowa City, Iowa, Iowa, United States; Healthlinx, DAVENPORT, Iowa, United States; University of Iowa College of Nursing, Iowa City, Iowa, United States; University of Iowa, Iowa City, Iowa, United States; Geroge Washington University, Arlington, Virginia, United States; George Washington University, Washington, DC, District of Columbia, United States

## Abstract

Despite improvements in HIV treatment, nursing care for older adults living with HIV remains insufficiently tailored to their unique needs. This study aimed to identify and validate nursing diagnoses, interventions, and outcomes relevant to this population. A content validation survey was conducted with 27 HIV/AIDS nursing experts who evaluated 45 nursing diagnoses, 79 outcomes, and 82 interventions on their relevance using a five-point scale. Relevance ratios classified each item as critical, supplemental, or unnecessary. Experts identified critical nursing diagnoses such as risk for loneliness, impaired memory, and risk for infection. Key interventions included Medication Management, education on prescribed medications, and Emotional Support. Important nursing outcomes identified were Social Support, Infection Severity, and Immunization Behavior. These validated elements provide an evidence-based framework for developing holistic, individualized care plans addressing both HIV-specific and aging-related health concerns. Incorporating these findings into clinical nursing practice can improve assessment, planning, and care delivery for older adults living with HIV. Nurses are encouraged to use these validated care components to deliver comprehensive, person-centered care that attends to physical, cognitive, and psychosocial needs, thereby enhancing health outcomes and quality of life for this growing population.

